# Meniscal forces and knee kinematics are affected by tibial slope modifying high tibial osteotomy

**DOI:** 10.1002/ksa.12577

**Published:** 2025-01-05

**Authors:** Philipp W. Winkler, Calvin K. Chan, Sene K. Polamalu, Gian Andrea Lucidi, Nyaluma N. Wagala, Jonathan D. Hughes, Richard E. Debski, Volker Musahl

**Affiliations:** ^1^ Orthopaedic Robotics Laboratory, Departments of Bioengineering and Orthopaedic Surgery University of Pittsburgh Pittsburgh Pennsylvania USA; ^2^ Department for Orthopaedics and Traumatology, Kepler University Hospital GmbH Johannes Kepler University Linz Linz Austria; ^3^ Department for Orthopaedic Sports Medicine, Klinikum rechts der Isar Technical University of Munich Munich Germany; ^4^ IIa Clinica, IRCCS Istituto Ortopedico Rizzoli Bologna Italia; ^5^ Department of Orthopaedic Surgery, UPMC Freddie Fu Sports Medicine Center University of Pittsburgh Pittsburgh Pennsylvania USA

**Keywords:** biomedical engineering, knee joint, meniscus, sports medicine, tibia

## Abstract

**Purpose:**

To quantify the effect of increasing the posterior tibial slope (PTS) on knee kinematics and the resultant medial and lateral meniscal forces.

**Methods:**

In this controlled laboratory study, a 6 degrees of freedom (DOF) robotic testing system was used to apply external loading conditions to seven fresh‐frozen human cadaveric knees: (1) 200‐N axial compressive load, (2) 5‐N m internal tibial +10‐N m valgus torque and (3) 5‐N m external tibial + 10‐N m varus torque. Knee kinematics and the resultant medial and lateral meniscal forces were acquired for two PTS states: (1) native PTS and (2) increased PTS. Resultant forces in the medial and lateral meniscus were calculated using the principle of superposition.

**Results:**

In response to 5‐N m external tibial + 10‐N m varus torque, significantly more internal tibial rotation was observed after increasing PTS at 60° (*p* = 0.0156) and 90° (*p* = 0.0156) flexion. Increasing PTS caused significantly more medial tibial translation from 30° to 90° flexion in response to 5‐N m internal tibial + 10‐N m valgus torque. In response to 5‐N m external tibial + 10‐N m varus torque, the resultant force in the medial meniscus at 60° flexion decreased significantly after increasing PTS (32.8%, *p* = 0.016). Resultant forces in the lateral meniscus decreased significantly after increasing PTS at 30° (34.5%; *p* = 0.016) and 90° (29.7%; *p* = 0.031) flexion in response to 5‐N m internal tibial + 10‐N m valgus torque.

**Conclusion:**

Increasing PTS in a native knee with intact cruciate ligaments affected 6 DOF knee kinematics and decreased resultant forces in the medial and lateral meniscus by up to 35% in response to combined rotatory loads. Therefore, increasing PTS during high tibial osteotomy in a knee with intact cruciate ligaments does not increase the force carried by the entire meniscus at time zero.

**Level of Evidence:**

N/A.

**Abbreviations**
ACL: anterior cruciate ligament
DOF: degrees of freedom
ETT: external tibial torque
ICC: intraclass correlation coefficient
ITT: internal tibial torque
N/A: not applicable
OA: osteoarthritis
PCL: posterior cruciate ligament
PTS: posterior tibial slope
UFS: universal force/moment sensor
ValTT: valgus tibial torque
VarTT: varus tibial torque

## INTRODUCTION

Anisotropic material properties combined with a sophisticated microscopic architecture make the human menisci indispensable components of a properly functioning knee joint [27]. Biomechanical research has shown that depending on the loading condition and knee flexion angle, 44%–78% and 70%–81% of the corresponding tibiofemoral compressive load is transmitted by the medial and lateral meniscus, respectively [11, 40, 43]. Considering the commonly proposed radial zones of the menisci (anterior horn, midbody and posterior horn) [25], the majority of the compressive load of the medial compartment is transmitted by the posterior horn of the medial meniscus [43]. However, the posterior tibial slope (PTS), which has been shown to affect kinematics and contact mechanics of the tibiofemoral joint, has not been considered in such investigations [1, 10, 16, 19, 32, 48].

Several biomechanical studies have demonstrated that increasing PTS by 4–20° significantly increases anterior and proximal tibial translation relative to the femoral condyles [1, 10, 15, 16]. Moreover, one study has shown that increasing PTS resulted in a significant anterior shift of the tibiofemoral contact area, thereby unloading the posterior part of the tibial plateau [1]. Consequently, it can be assumed that the posterior horns of the menisci get unloaded, resulting in lower forces acting on the meniscus tissue. Therefore, the menisci may be incapable of their native function (i.e., force transmission and stabilization) after increasing PTS. Unloading the posterior horns of the menisci may reduce their stabilizing effect, resulting in increased translational and rotatory knee laxity [30, 34]. Also, an anterior shift of the tibiofemoral contact area inevitably changes the pressure distribution across the articular cartilage, potentially increasing the risk for the development of knee osteoarthritis (OA) [1].

So far, biomechanical research with respect to the PTS has primarily focused on knee kinematics and anterior cruciate ligament (ACL) and posterior cruciate ligament (PCL) graft forces [5, 6, 20, 39, 48]. As a result, PTS‐changing osteotomies are becoming increasingly more utilized, as reflected in good clinical and functional outcomes [2, 9, 41, 42]. Many knees indicated for PTS modification have advanced changes of the articular cartilage and the menisci, however, little is known about the effect of PTS modification on meniscal forces.

The purpose of this study was to quantify the effect of increasing PTS on knee kinematics and the resultant medial and lateral meniscal forces in human cadaveric knees using a 6 degrees of freedom (DOF) robotic testing system. It was hypothesized that increasing PTS would increase anterior and proximal tibial translation and decrease the resultant medial and lateral meniscal forces.

## METHODS

Seven fresh‐frozen cadaveric human knee specimens with a mean age of 51.9 ± 19.8 years (range, 21–75 years; 29% female) were included in this biomechanical study. Approval for this study was obtained by the institutional review board of the University of Pittsburgh (CORID #331).

Specimen preparation, surgical procedures, the experimental setup and the biomechanical testing protocol have been extensively reported in a previously published study [45]. The key steps and most important methodology are briefly summarized below.

Before being tested, the specimens were thawed for 24 h at room temperature. All specimens were examined manually, fluoroscopically and arthroscopically prior to testing. Exclusion criteria included a PTS greater than 12°, ligament or meniscus injuries, cartilage defects >Grade 2 according to the International Cartilage Repair Society grading system [7], and OA >Grade 2 based on the Kellgren–Lawrence grading scale [22].

Both the femur and the tibia were cut 20 cm proximal and distal to the joint line. Soft tissue 10 cm proximal and distal to the joint line was removed and the fibula was screwed to the tibia before the femoral and tibial bones were potted using an epoxy compound (Bondo; 3 M).

### PTS states

Each specimen was tested in two different PTS states: (1) osteotomized knee with native PTS and (2) osteotomized knee with increased PTS. The PTS states were evaluated after performing the osteotomy. Consequently, any influence of the surgical approach on the main outcome measures (knee kinematics and resultant meniscal forces) could be avoided. Therefore, the identified changes in knee kinematics and resultant forces in the medial and lateral meniscus were exclusively attributable to the alteration of the PTS.

**Figure 1:**
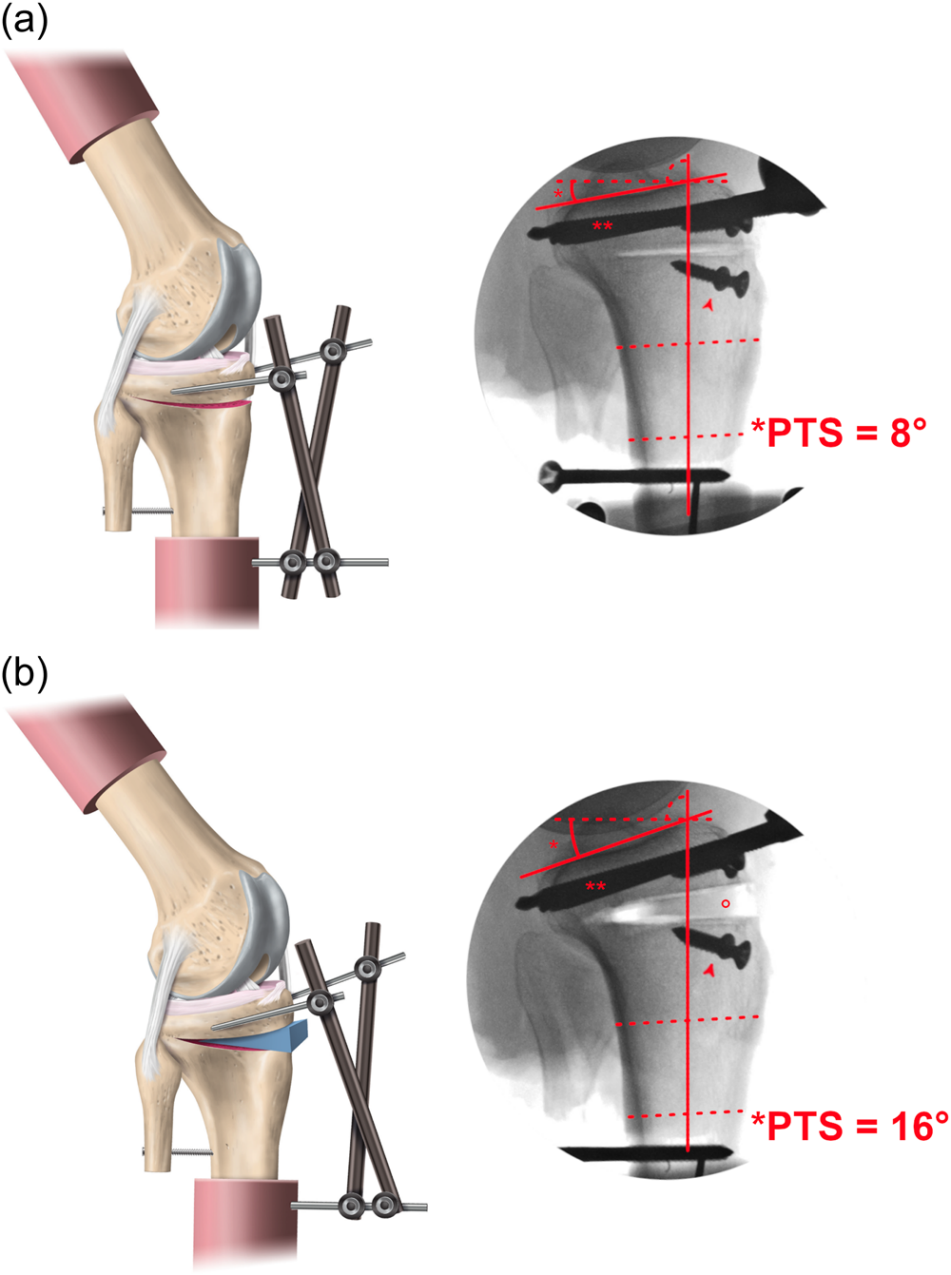
Schematic and fluoroscopic illustration of the posterior tibial slope (PTS) states. Osteotomized native PTS state (a) and increased PTS state (b). Rods proximal (**) and distal to the osteotomy gap were used to secure the osteotomy with an external fixator. The distance between screws (arrowhead) proximal and distal to the osteotomy gap was measured for repeatable PTS adjustment. °, epoxy compound wedge (blue) wedge within the osteotomy gap. (Reprinted and modified with permission from Winkler et al. [45]).

After potting, an external fixator (Hoffmann 3 Modular External Fixation; Stryker GmbH) was fixed to the tibial bone in a triangular shape. Consequently, the rigid transmission of forces along the proximal anatomic axis of the tibia without affecting the outcome measures was ensured (Figure 1) [6, 19, 20, 39].

A skin incision medial and lateral to the tibial tubercle was made. Two K‐wires were placed in the proximal tibia under fluoroscopic guidance to guide the osteotomy. A supra‐tuberositary osteotomy was performed using an oscillating saw (saw blade thickness, 2 mm). Care was taken not to damage the joint capsule and the capsuloligamentous structures of the knee. The osteotomy ended 5 mm anterior to the posterior tibial cortex next to the tibial PCL insertion site. Care was taken to preserve the posterior cortical hinge in order to reliably change the PTS. The intact posterior cortical hinge was confirmed using fluoroscopy.

After gradually increasing the osteotomy gap, an epoxy compound wedge (custom‐made, wedge base height, 10 mm) was inserted in the osteotomy gap with the base of the wedge touching the anterior cortex of the tibia and the external fixator was re‐secured. As a result, the anterior edge of the osteotomy was opened by 10 mm, leading to a change in PTS depending on the knee size [10, 15]. To reduce the PTS, the steps were reversed. After each PTS modification, the wound was closed in layers.

### Experimental setup and protocol

Specimens were fixed to a 6 DOF robotic testing system (MJT model FRS2010). A universal force/moment sensor (UFS; ATI Delta IP60 model SI‐660‐60) provided feedback to the controller. A LabView Programme (Technology Services Inc.) controlled the robotic testing system. The translational and rotational position repeatability of the robotic testing system was shown to be less than ±0.015 mm and ±0.01°, respectively. The measurement uncertainty of the UFS was found to be approximately 1% of the full scale [4]. The robotic testing system used in this study was specifically designed to assess knee biomechanics and has a higher accuracy than many other robotic systems used in previous biomechanical research [8, 17].

The femoral insertion sites of the collateral ligaments were used to define the medial‐lateral translation axis and flexion‐extension rotation axis. The proximal tibial shaft axis was used to define the proximal‐distal translation axis and internal‐external rotation axis. The cross‐product of the two mentioned axes defined the anterior‐posterior translation axis and varus‐valgus rotation axis [18].

The passive path from full extension (i.e., 1‐N m extension moment) to 90° of knee flexion of the osteotomized intact knee with the native PTS state (i.e., external fixator attached and osteotomy performed) was determined. Minimized forces and moments across all axes of the coordinate system over the entire range of motion were needed to obtain the passive path of flexion‐extension [13, 14, 35, 38]. The following loading conditions were applied to the osteotomized knee with the native PTS state: (1) 200‐N axial compressive load, (2) 5‐N m internal tibial torque combined with 10‐N m valgus tibial torque and (3) 5‐N m external tibial torque combined with 10‐N m varus tibial torque. During load application, the knee was flexed from full extension to 90° flexion and the knee kinematics were recorded. Previous studies have shown that such loading conditions cause stress on the menisci and are suitable to evaluate resultant meniscal forces [34, 35]. To account for any effects of viscoelasticity, all loading conditions were repeated five times. For final analysis, the data from the last cycle were used. After recording the knee kinematics of the osteotomized intact knee with the native PTS state, the PTS was increased, and the knee kinematics for the osteotomized intact knee with the increased PTS state were recorded in the same manner for all three loading conditions.

Next, the medial and lateral meniscus were arthroscopically removed sequentially. In the medial meniscus deficient and in the medial and lateral meniscus deficient knee, the previously recorded 6 DOF kinematics for each loading condition were repeated in both PTS states using a position‐control mode. The new forces and moments for each knee condition were measured by the UFS, and the resultant forces in the medial and lateral meniscus were calculated for both PTS states by the principle of superposition (Table 1) [12, 26]. Calculating the resultant forces in the menisci using the principle of superposition is linked to the high accuracy of the robotic testing system and has been proven in previous studies [31, 34, 35]. In the past, Fuji Film or TekScan systems were used to assess meniscus function by measuring contact area and contact pressure with limited accuracy [3]. Hydration of the specimens was ensured during testing [46].

**Table 1:** Experimental protocol and acquired data.

Knee condition, PTS state	Loading condition/replay kinematics^a^	Data acquired
Intact, osteotomized native PTS	(1) 200‐N Axial compression (2) 5‐N m ITT + 10‐N m ValTT (3) 5‐N m ETT + 10‐N m VarTT	(a) Kinematics of intact knee with osteotomized native PTS
Intact, increased PTS	(1) 200‐N Axial compression (2) 5‐N m ITT + 10‐N m ValTT (3) 5‐N m ETT + 10‐N m VarTT	(b) Kinematics of intact knee with increased PTS
Medial meniscus deficient, osteotomized native PTS	Replay recorded kinematics of (a)	Resultant force: medial meniscus with osteotomized native PTS
Medial meniscus deficient, increased PTS	Replay recorded kinematics of (b)	Resultant force: medial meniscus with increased PTS
Medial + lateral meniscus deficient, increased PTS	Replay recorded kinematics of (b)	Resultant force: lateral meniscus with increased PTS
Medial + lateral meniscus deficient, osteotomized native PTS	Replay recorded kinematics of (a)	Resultant force: lateral meniscus with osteotomized native PTS

Abbreviations: ETT, external tibial torque; ITT, internal tibial torque; PTS, posterior tibial slope; ValTT, valgus tibial torque; VarTT, varus tibial torque.

^a^ Replay (a) and (b) refers to reproduction of previously recorded kinematics of (a) intact knee with osteotomized native PTS and (b) intact knee with increased PTS.

### Repeatability of multiple PTS adjustments

The medial PTS was measured using the proximal anatomic axis of the tibia and a line tangential to the medial tibial plateau using strict lateral radiographs (Figure 1) [21]. PTS measurements were performed by the main observer (PWW). Inter‐ and intra‐rater reliability of PTS measurements was assessed by intraclass correlation coefficients (ICCs). Posterior tibial slope measurements were performed on radiographs of 17 human knee specimens three times (2‐week intervals) by observer one (PWW) and once by observer two (NNW) and three (JDH). Excellent intra‐rater reliability (ICC, 0.994; 95% confidence interval [CI]: 0.984–0.998) and good to excellent inter‐rater reliability (ICC, 0.916; 95% CI: 0.755–0.977) were found for medial PTS measurements [24]. Measurements were performed using ImageJ version 1.52a (Wayne Rasband, National Institutes of Health).

In order to apply the principle of superposition reliably, the two PTS states had to be reproduced precisely several times to calculate the resulting forces of the menisci in the two PTS states within the same specimen. Good to excellent repeatability of multiple PTS adjustments with an accuracy of ±0.2° and measurements of the resultant forces in the menisci within the repeatability of the robotic testing system have been found in preliminary tests.

Reliable multiple PTS adjustments were achieved by a three‐step protocol for each modification of PTS: (1) Both PTS states were marked on the external fixator to ensure that the position of the pin‐to‐rod couplings could be accurately reproduced for each PTS state. (2) PTS measurement was performed after each PTS modification. The PTS had to be within ±0.2° of the corresponding PTS state to continue the testing protocol. (3) Reference screws were fixed on both sides of the patellar tendon, both proximal and distal of the osteotomy (Figure 1). The distances between the proximal and distal reference screws (medial and lateral) were measured using a calliper after PTS modification, resulting in a repeatability of ±0.1 mm of distance restoration.

### Statistical analysis

Sample size calculation was performed using G*Power (Erdfelder, Faul, Buchner, Lang, HHU Düsseldorf) and was based on preliminary tests. A minimum of four cadaveric knees were required to detect significant differences in resultant forces in the medial meniscus between the two PTS states (effect size, 2.77; *ɑ*, 0.05; power, 0.8).

Data distribution was assessed by the Shapiro–Wilk test. Continuous data were normally distributed and expressed as mean, standard deviation, and range. Knee kinematics were reported as the difference between the passive path of motion and the path of motion during load application. A repeated measures analysis of variance was conducted to assess the effects of the factors ‘PTS’ (osteotomized native vs. osteotomized increased) and ‘knee flexion angle’ (full extension vs. 30° vs. 60° vs. 90°) and a potential PTS × knee flexion angle interaction on knee kinematics and the resultant forces in the medial and lateral meniscus and followed by Tukey post hoc tests. SPSS software version 26.0 (IBM‐SPSS) was used for statistical analysis. The level of significance was defined as *p* < 0.05.

## RESULTS

The native PTS was 9.1 ± 2.3° (range, 8.0–11.1°). No difference between the native PTS and the PTS of the osteotomized knee in the native PTS state was found (9.2 ± 2.2° [range, 8.0–11.2°]; *p* > 0.050). A statistically significant increase in PTS (8.6°) after performing the anterior open wedge osteotomy was found (osteotomized native PTS state, 9.2 ± 2.2° vs. osteotomized increased PTS state, 17.8 ± 1.7°; *p* < 0.001).

### Knee kinematics

In response to 200‐N axial compressive load, a significant increase in anterior tibial translation existed at 30° (4.5 ± 1.4 mm vs. 2.6 ± 2.2 mm, *p* = 0.016), 60° (2.6 ± 1.1 mm vs. 0.9 ± 1.1 mm, *p* = 0.016) and 90° (1.1 ± 0.8 mm vs. 0.1 ± 0.8 mm, *p* = 0.016) knee flexion after increasing the PTS. In addition, proximal tibial translation was significantly higher in the increased PTS state compared to the native PTS state at 30° flexion in response to 200‐N axial compressive load (2.1 ± 0.7 mm vs. 1.6 ± 0.4 mm, *p* = 0.031). In response to 5‐N m external tibial + 10‐N m varus torque increasing PTS caused overall less anterior tibial translation and significantly more lateral tibial translation from full extension to 90° flexion. In addition, significantly more varus tibial rotation was observed after increasing PTS at full extension (2.2 ± 1.0° vs. 3.3 ± 1.0°, *p* = 0.0156), 30° (2.9 ± 1.3° vs. 4.5 ± 1.2°, *p* = 0.156), and 90° (5.8 ± 1.6° vs. 6.9 ± 1.6°, *p* = 0.0313) flexion in response to 5‐N m external tibial + 10‐N m varus torque. Significantly more internal tibial rotation was observed after increasing PTS at 60° (16.9 ± 2.3° vs. 18.6 ± 2.0°, *p* = 0.0156) and 90° (16.7 ± 2.6° vs. 19.1 ± 2.0°, *p* = 0.0156) flexion in response to 5‐N m external tibial + 10‐N m varus torque. Increasing PTS caused overall less anterior tibial translation and significantly more medial tibial translation from 30° to 90° flexion in response to 5‐N m internal tibial + 10‐N m valgus torque. In addition, significantly more valgus tibial rotation was observed from full extension to 90° flexion after increasing PTS in response to 5‐N m internal tibial + 10‐N m valgus torque. The kinematic data are presented in further detail in Supporting Information S1: Supplement 1.

### Resultant force in the medial meniscus

**Figure 2:**
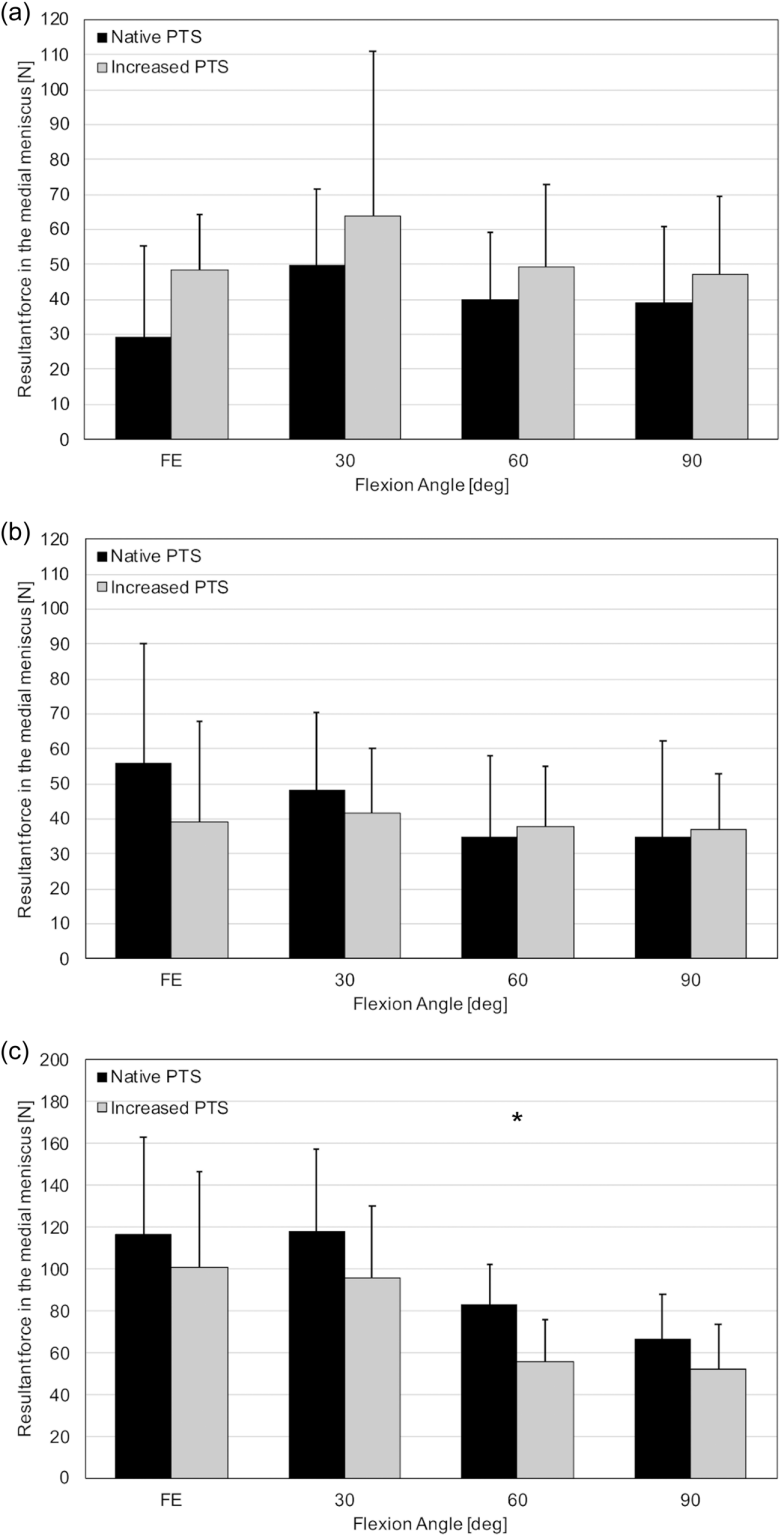
Resultant force in the medial meniscus. Resultant force in the medial meniscus versus flexion angle in the native and increased posterior tibial slope (PTS) state in response to 200‐N axial compressive load (a), 5‐N m internal tibial torque + 10‐N m valgus tibial torque (b), and 5‐N m external tibial torque + 10‐N m varus tibial torque (c). *, Statistically significant difference between the native and increased PTS state (*p* < 0.05).

Overall, resultant forces in the medial meniscus were highest in response to 5‐N m external tibial + 10‐N m varus torque, followed by 200‐N axial compressive load, and 5‐N m internal tibial + 10‐N m valgus torque. In response to 5‐N m external tibial + 10‐N m varus torque, the resultant force in the medial meniscus at 60° flexion decreased significantly after increasing PTS (32.8%, 83.2 ± 19.1 N vs. 55.9 ± 19.9 N, *p* = 0.016). No differences in the resultant forces in the medial meniscus after increasing PTS were observed in response to 200‐N axial compressive load and 5‐N m internal tibial + 10‐N m valgus torque (Figure 2).

### Resultant force in the lateral meniscus

**Figure 3:**
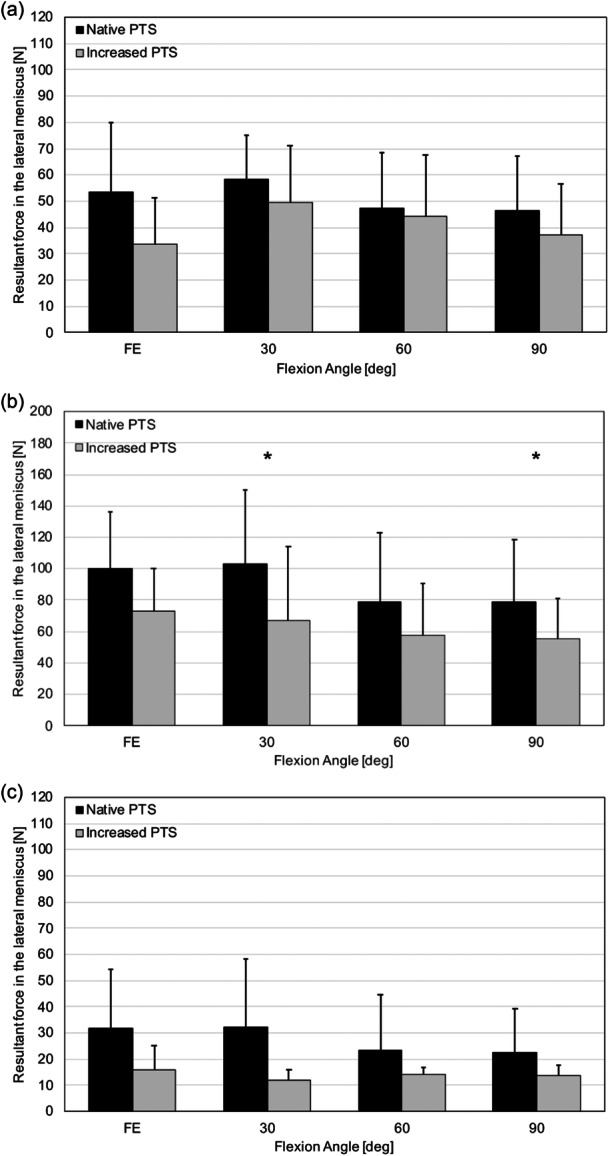
Resultant force in the lateral meniscus. Resultant force in the lateral meniscus versus flexion angle in the native and increased posterior tibial slope (PTS) state in response to 200‐N axial compressive load (a), 5‐N m internal tibial torque + 10‐N m valgus tibial torque (b), and 5‐N m external tibial torque + 10‐N m varus tibial torque (c). *, Statistically significant difference between the native and increased PTS state (*p* < 0.05).

Overall, resultant forces in the lateral meniscus were highest in response to 5‐N m internal tibial + 10‐N m valgus torque, followed by 200‐N axial compressive load and 5‐N m external tibial + 10‐N m varus torque. Resultant forces in the lateral meniscus decreased significantly after increasing PTS at 30° (34.5%; 103.0 ± 47.3 N vs. 67.5 ± 46.4 N; *p* = 0.016) and 90° (29.7%; 78.9 ± 39.5 N vs. 55.5 ± 25.2 N; *p* = 0.031) flexion in response to 5‐N m internal tibial + 10‐N m valgus torque. No differences in the resultant forces in the lateral meniscus after increasing PTS were observed in response to 200‐N axial compressive load and 5‐N m external tibial + 10‐N m varus torque (Figure 3).

## DISCUSSION

The most important finding of this study was that increasing PTS in a native knee with intact cruciate ligaments affected 6 DOF knee kinematics and decreased resultant forces in the medial and lateral meniscus in response to combined rotatory loads. Based on the results of this study, intentionally or unintentionally increasing PTS during high tibial osteotomy in a knee with intact cruciate ligaments does not increase the force carried by the entire meniscus at time zero.

Several observational clinical studies indicate an association between meniscus tears and PTS. A recent meta‐analysis evaluating risk factors for lateral meniscus posterior root tears in patients with ACL injury analysed over 6000 patients from 17 studies. It was shown that amongst other demographic and radiographic factors, high lateral PTS was associated with an increased risk of lateral meniscus posterior root tears [47]. Similarly, high PTS has been associated with an increased risk of medial meniscus posterior horn and posterior root tears in several studies [23, 29, 36]. Such observations could be explained by the biomechanically confirmed increased anterior tibial translation in knees with increased PTS. In this study, PTS‐increasing osteotomy increased anterior tibial translation by up to 1.8 mm. This is consistent with previous controlled laboratory studies that have found an increase in anterior tibial translation of 2–7 mm after an increase in PTS at various loading conditions, PTS states, and flexion angles [1, 15, 16]. Note that in this study, the force carried by the entire meniscus (i.e., resultant force in the meniscus) was determined. This may explain why the risk of injury in some meniscal areas was associated with increased PTS in previous studies, but the resultant force in the menisci decreased with increasing PTS in this study.

To date, biomechanical studies evaluating the association between PTS and the forces acting on the menisci are limited. In a recent study, shear forces and compression on the medial meniscus posterior root were evaluated at various PTS states using ten cadaveric knee specimens attached to a uniaxial materials testing machine. Increasing PTS caused increased compression and anterior shear force at the medial meniscus posterior root. The authors suggest that the reported results may assist in decision‐making for PTS‐modifying osteotomy in the setting of meniscus root or ACL injury [28]. These findings are different compared to the results of the present study, where decreased resultant forces in the medial and lateral meniscus were observed after increasing the PTS. These inconsistent findings can be attributed to several methodological aspects. In the present study, resultant forces in the menisci were determined by the principle of superposition, without differentiating between the specific meniscus areas, while forces acting on the meniscus root were previously determined by a load cell attached to the medial meniscus posterior root [28]. The resultant forces in a meniscus, as determined in the present study, reflect the entire force carried by the respective meniscus [33, 35]. In contrast, meniscus forces measured by a load cell are limited to the meniscus root disregarding the remaining regions of the meniscus. Moreover, different loading conditions and DOF were used in the present study compared to the previous study [28], impeding a reliable comparison.

As demonstrated by a previous biomechanical investigation, the posterior horn of the meniscus has the highest contribution to force transmission, especially at higher flexion angles [43]. Considering the importance of the posterior meniscus horn in force transmission, a decrease in resultant forces in the menisci of up to 35% in the increased compared to the native PTS state would indicate unloading of the posterior meniscus horn caused by PTS‐increasing osteotomy. This assumption was confirmed by a biomechanical study in which tibiofemoral contact mechanics were investigated after PTS‐increasing osteotomy in seven intact human cadaveric knees using a knee simulator [1]. Using pressure‐sensitive films, increasing the PTS in 5° increments caused a gradual anterior shift of the tibiofemoral contact area and contact pressure on the tibial plateau during an isokinetic extension‐flexion motion. Increasing the PTS to 20° decreased the load carried by the posterior half of the tibial plateau by 36% compared to the native PTS state [1]. The authors suggested that PTS‐increasing osteotomy in an ACL‐ and PCL‐intact knee results in decompression of the posterior part of the tibial plateau caused by an anterior and proximal translation of the tibia relative to the femoral condyles [1]. This finding agrees with the kinematic data in the current study and supports the finding of decreased resultant forces in the menisci after increasing the PTS. Lower resultant forces in the menisci caused by anterior and proximal tibial translation may indicate the unloading of the posterior horns of the menisci. This might be beneficial after meniscal repair or to unload degenerated cartilage after partial meniscectomy often observed during high tibial osteotomy.

Some limitations of this study should be mentioned. In this study, the resultant forces in the menisci were determined reflecting the total force carried by the respective meniscus. Therefore, it was not possible to determine the forces acting on specific areas of the meniscus, such as the posterior meniscus root or the posterior meniscus horn, which are most commonly affected by injuries. In addition, the stiffness of the entire construct (knee specimen with external fixator) was not assessed, which could have affected the forces determined by the principle of superposition [37]. Moreover, the applied loading conditions have been proven in biomechanical research, but do not reflect daily activities. Higher loads may have been more efficient in detecting changes in meniscal forces. Another limitation was that only the medial PTS was measured by fluoroscopy, although differences between the medial and lateral PTS have been reported [44].

## CONCLUSIONS

In this biomechanical study, increasing PTS in a native knee with intact cruciate ligaments affected 6 DOF knee kinematics and decreased resultant forces in the medial and lateral meniscus by up to 35% in response to combined rotatory loads, while no difference was observed in response to 200‐N axial compressive load. Consequently, intentionally or unintentionally increasing PTS during high tibial osteotomy in a knee with intact cruciate ligaments does not increase the force carried by the entire meniscus at time zero.

## AUTHOR CONTRIBUTIONS

All listed authors have contributed substantially to this work: Philipp W. Winkler, Nyaluma N. Wagala, Gian Andrea Lucidi, Calvin K. Chan, Sene K. Polamalu and Jonathan D. Hughes collected data, performed statistical analysis, literature review and primary manuscript preparation. Volker Musahl and Richard E. Debski assisted with interpretation of the results, initial drafting of the manuscript, as well as editing and final manuscript preparation. All authors read and approved the final manuscript.

## CONFLICT OF INTEREST STATEMENT

Volker Musahl reports educational grants, consulting fees and speaking fees from Newclip, Smith & Nephew plc, educational grants from Arthrex and Smith & Nephew, is a board member of the International Society of Arthroscopy, Knee Surgery and Orthopaedic Sports Medicine (ISAKOS), and deputy editor‐in‐chief of Knee Surgery, Sports Traumatology, Arthroscopy (KSSTA). The remaining authors declare no conflicts of interest.

## ETHICS STATEMENT

This study was approved by the Institutional Review Board of the University of Pittsburgh (CORID #331).

## Supporting information

Supporting information.

## Data Availability

Data are available from the corresponding author upon reasonable request.
