# Supplementary material for: Meniscal forces and knee kinematics are affected by tibial slope modifying high tibial osteotomy
Source: Knee Surg Sports Traumatol Arthrosc. 2025 Jan 5;33(7):2345–55. doi: 10.1002/ksa.12577 (PMC12205423; doi:10.1002/ksa.12577)
Supplement: Supplementary file 1 — Supporting information. [file KSA-33-2345-s001.docx]

**Supplement 1. Knee kinematics in the native and increased PTS state for each loading condition and flexion angle.**

| **Loading Condition** | **Kinematics** | **Native PTS state** | | | | **Increased PTS state** | | | |
| --- | --- | --- | --- | --- | --- | --- | --- | --- | --- |
|  |  | FE | 30° | 60° | 90° | FE | 30° | 60° | 90° |
| 200-N Axial compression | A-P, [mm] | 1.4 ± 0.8 | 2.6 ± 2.2 | 0.9 ± 1.1 | 0.1 ± 0.8 | 1.4 ± 0.6 | 4.5 ± 1.4* | 2.6 ± 1.1* | 1.1 ± 0.8* |
|  | P-D, [mm] | 1.0 ± 0.1 | 1.6 ± 0.4 | 2.2 ± 0.5 | 2.7 ± 0.7 | 1.0 ± 0.2 | 2.1 ± 0.7* | 2.7 ± 1.1 | 2.9 ± 1.1 |
|  | M-L, [mm] | 0.0 ± 0.5 | -0.6 ± 1.3 | -0.2 ± 2.0 | 0.3 ± 2.9 | 0.2 ± 0.4* | -1.4 ± 1.5* | -1.3 ± 2.3* | 0.0 ± 3.2 |
|  | IR-ER, [deg] | 1.0 ± 2.6 | 4.5 ± 5.8 | 4.1 ± 7.8 | 2.9 ± 8.7 | 0.0 ± 0.9 | 4.9 ± 3.9 | 3.9 ± 4.6 | 1.2 ± 5.9 |
|  | V-V, [deg] | 0.1 ± 0.4 | 0.6 ± 0.9 | 0.5 ± 1.1 | 0.5 ± 1.3 | 0.1 ± 0.4 | 1.6 ± 1.4* | 2.0 ± 2.0* | 1.3 ± 1.9 |
| 5-Nm ITT + 10-Nm ValTT | A-P, [mm] | 1.9 ± 1.4 | 4.0 ± 1.6 | 0.9 ± 1.4 | -0.4 ± 1.5 | 0.3 ± 0.6* | 3.1 ± 1.7 | 0.6 ± 1.0 | -1.2 ± 0.8 |
|  | P-D, [mm] | -0.5 ± 0.9 | 0.1 ± 1.0 | -0.1 ± 1.5 | -0.6 ± 1.8 | -0.3 ± 0.4 | 0.5 ± 1.3 | 0.1 ± 2.3 | -0.7 ± 2.4 |
|  | M-L, [mm] | -3.5 ± 1.5 | -5.7 ± 2.3 | -6.2 ± 2.3 | -6.9 ± 2.3 | -4.1 ± 2.1 | -7.0 ± 3.2* | -8.2 ± 3.3* | -7.8 ± 2.6* |
|  | IR-ER, [deg] | 13.4 ± 4.2 | 20.8 ± 3.4 | 21.5 ± 4.5 | 20.7 ± 4.4 | 12.5 ± 4.7 | 20.9 ± 4.6 | 21.3 ± 3.9 | 19.2 ± 2.5 |
|  | V-V, [deg] | 3.6 ± 1.1 | 5.8 ± 1.3 | 6.6 ± 1.4 | 7.5 ± 1.3 | 4.3 ± 1.7* | 7.8 ± 2.3* | 9.5 ± 2.2* | 9.5 ± 1.9* |
| 5-Nm ETT + 10-Nm VarTT | A-P, [mm] | 0.9 ± 1.1 | 3.1 ± 0.9 | 0.7 ± 1.1 | 0.2 ± 1.3 | -0.2 ± 0.7 | 3.0 ± 1.2 | 0.3 ± 1.6 | -0.5 ± 1.4 |
|  | P-D, [mm] | -0.1 ± 0.3 | -0.2 ± 0.6 | -0.7 ± 0.8 | -1.0 ± 1.1 | -0.1 ± 0.4 | 0.0 ± 0.5 | -0.4 ± 0.6 | -0.9 ± 1.2 |
|  | M-L, [mm] | 2.3 ± 1.3 | 4.0 ± 1.3 | 5.0 ± 1.6 | 5.4 ± 1.7 | 3.4 ±1.5* | 5.9 ± 1.7* | 7.0 ± 2.1* | 7.8 ± 2.4* |
|  | IR-ER, [deg] | -14.2 ± 3.4 | -18.4 ± 3.0 | -16.9 ± 2.3 | -16.7 ± 2.6 | -10.8 ± 2.7* | -18.6 ± 3.1 | -18.6 ± 2.0 | -19.1 ± 2.0* |
|  | V-V, [deg] | -2.2 ± 1.0 | -2.9 ± 1.3 | -4.6 ± 1.7 | -5.8 ± 1.6 | -3.3 ± 1.0* | -4.5 ± 1.2* | -5.2 ± 1.7 | -6.9 ± 1.6* |

***Legend supplement 1:*** *A-P, anterior-posterior (positive values indicate anterior tibial translation); ETT, external tibial torque; FE, full extension (1-Nm extension moment); IR-ER, internal tibial rotation-external tibial rotation (positive values indicate internal tibial rotation); ITT, internal tibial torque; M-L, medial-lateral (positive values indicate lateral tibial translation); P-D, proximal-distal (positive values indicate proximal tibial translation); PTS, posterior tibial slope; V-V, varus tibial rotation-valgus tibial rotation (positive values indicate valgus tibial rotation); ValTT, valgus tibial torque; VarTT, varus tibial torque; *, statistically significant difference compared to the native PTS state (p < 0.05).*
